# Society for immunotherapy of cancer (SITC) statement on the proposed changes to the common rule

**DOI:** 10.1186/s40425-016-0142-0

**Published:** 2016-06-21

**Authors:** Howard L. Kaufman, Lisa H. Butterfield, Pierre G. Coulie, Sandra Demaria, Robert L. Ferris, Jérôme Galon, Samir N. Khleif, Ira Mellman, Pamela S. Ohashi, Willem W. Overwijk, Suzanne L. Topalian, Francesco M. Marincola

**Affiliations:** Rutgers Cancer Institute of New Jersey, 195 Little Albany Street, Room 2004, New Brunswick, NJ 08901 USA; University of Pittsburgh, Pittsburgh, PA USA; De Duve Institute, Universite Catholique De Louvain, Brussels, Belgium; Weill Cornell Medicine, New York, NY USA; Cordeliers Research Center, INSERMFrench National Institute of Health and Medical Research, Paris, France; Georgia Regents University Cancer Center, Augusta, GA France; Genentech, Inc., San Francisco, CA USA; Princess Margaret Cancer Centre, University Health Network, Toronto, Canada; MD Anderson Cancer Center, University of Texas, Houston, TX USA; Johns Hopkins University School of Medicine, Baltimore, MD USA; Sidra Medical Center, Doha, Qatar

## Abstract

The Common Rule is a set of ethical principles that provide guidance on the management of human subjects taking part in biomedical and behavioral research in the United States. The elements of the Common Rule were initially developed in 1981 following a revision of the Declaration of Helsinki in 1975. Most academic facilities follow the Common Rule in the regulation of clinical trials research. Recently, the government has suggested a revision of the Common Rule to include more contemporary and streamlined oversight of clinical research. In this commentary, the leadership of the Society for Immunotherapy of Cancer (SITC) provides their opinion on this plan. While the Society recognizes the considerable contribution of clinical research in supporting progress in tumor immunotherapy and supports the need for revisions to the Common Rule, there is also some concern over certain elements which may restrict access to biospecimens and clinical data at a time when high throughput technologies, computational biology and assay standardization is allowing major advances in understanding cancer biology and providing potential predictive biomarkers of immunotherapy response. The Society values its professional commitment to patients for improving clinical outcomes with tumor immunotherapy and supports continued discussion with all stakeholders before implementing changes to the Common Rule in order to ensure maximal patient protections while promoting continued clinical research at this historic time in cancer research.

There has been considerable progress in the development of immunotherapy for the treatment of cancer over the last decade. This has been possible because of advances in our understanding of the basic mechanisms that govern tumor immunity and T cell regulation, commitment from the pharmaceutical and biotechnology industry to drug development in immunotherapy, and the coordinated completion of well-designed clinical trials [1, 2]. The importance of immunotherapy is highlighted by the occurrence of durable response rates in some patients and evidence that immunotherapy may have therapeutic activity in a large number of cancers. Furthermore, emerging data in melanoma support a role for combination immunotherapy as potentially more effective than monotherapy [3]. Future priorities include understanding mechanisms of resistance to immunotherapy and identification of predictive biomarkers that will help select appropriate patients for treatment and allow more efficient clinical monitoring of anti-tumor responses in patients on specific regimens.

A major reason for the success of immunotherapy in changing the treatment paradigm for cancer has been the participation of cancer patients in clinical trials. As experts in the field, we have a responsibility to ensure that patients are provided with appropriate information to make a truly informed decision about clinical trial participation. This process is complex because of the often challenging prospect of balancing potential (or even unknown) benefit, availability of alternative treatment options, and the possibility of experiencing adverse events, or more succinctly stated: the need to balance the hope and the reality.

The informed consent process allows patients, or their responsible proxy, to provide voluntary agreement to participate in a clinical study after being informed of the study purpose, the experimental drugs or procedures involved, the therapeutic alternatives, the potential for personal or societal benefit, the potential type and intensity of risks (including lack of response and adverse events) and information on sponsors and healthcare provider conflict of interest. The essential criteria of informed consent have been well described and require that potential study subjects have knowledge and comprehend the proposed study, that their consent is given freely without undue influence and that patients can withdraw consent at any time [4–6]. The many ethical issues associated with informed consent were summarized in the Belmont Report, which was developed after a conference at the Smithsonian Institution’s Belmont Conference Center in 1976, with additions from the National Commission for the Protection of Human Subjects of Biomedical and Behavioral Research in 1979 and the U.S. Food and Drug Administration in 1981 [7]. The Belmont Report provides guidelines for the ethical conduct of clinical research and considers the individual rights of patients to make informed decisions and considers respect for persons, beneficence and justice. These principles recognize that patients have the right to act as autonomous decision makers with respect to the treatment of their disease, special populations may require additional protections to avoid harm, patients are treated in a fair and equitable manner while entering into a treatment strategy that may have limited or unknown effects and that the selection of subjects and their clinical management be fair and balanced without coercion.

In order to ensure the principles set forth in the Belmont Report are followed in clinical research, the federal government in 1991 adopted the so-called Common Rule. The Common Rule is a federal policy that outlines how human subjects will be protected when electing participation in a clinical trial. The Common Rule requires compliance with contemporary ethical guidelines by research institutions, provides regulations for local Institution Review Board (IRB) review of clinical studies, and mandates the collection and documentation of informed consent [4]. While the Common Rule has provided guidance on how to conduct responsible clinical research for the last 25 years, there is currently interest in modifying the Common Rule in order to better protect research subjects and help promote public trust relating to the informed consent process [8].

Tumor immunotherapy may be one of the best examples of why the Common Rule should be amended. Some of the recent regulatory approvals of immunotherapy agents were based on data sets from early phase clinical trials [9–11]. This likely relates to improved understanding of tumor immunity and improved rationale for drugs entering the clinic, as well as recognition of areas of unmet clinical need. Thus, the likelihood of direct clinical benefit to a patient is higher today than it was 25 years ago. Further, there are now better guidelines in place for the management of immune-related adverse events. This has significantly shifted the benefit/risk ratio for participating in clinical research studies in the modern era. This shift needs to be fairly communicated to potential study subjects and the demonstration of benefit in cancers not traditionally characterized as “immunogenic” suggests that there may be a greater benefit to society for rapidly conducting and completing tumor immunotherapy research studies. While these developments are exciting, it is also important to maintain a fair and balanced perspective for potential human subjects while avoiding over-emphasizing any individual drug or therapeutic regimen.

The Society for Immunotherapy of Cancer (SITC) is the primary member-driven professional organization dedicated to improving outcomes for cancer patients through the implementation of immunotherapy. Many of our members are actively engaged in pre-clinical and clinical investigation of cancer immunotherapy and participate in the informed consent process. As a professional Society, we applaud the review of the Common Rule at this pivotal stage in the history of cancer research, and agree with many of the proposed changes. The changes that promote shorter documents with attention to the most meaningful information will help patients without medical backgrounds more easily understand and make decisions regarding clinical trials. We also support exclusion from the Common Rule of proposals that are not deemed to be research or that have limited risks to human subjects, which will allow less rigorous review by already overburdened IRBs and will speed the pace of scientific discovery. The ability to use a single IRB for cooperative group trials and changes to the continuing review process where subject harm is no longer possible, are also reasonable changes.

There are, however, some recommendations that are of particular concern to our community. Restrictions on the collection and testing of biomarkers may significantly hamper critical research in the field at a time when we are beginning to identify putative predictive biomarkers of immunotherapy response and resistance. The inclusion of high priority tumor, peripheral blood and bone marrow biopsies to investigate correlative endpoints in current immunotherapy studies have been critical in identifying underlying mechanisms of response and providing new biomarkers for validation [12]. This is well exemplified by the finding that intratumoral expression of programmed cell death ligand 1 (PD-L1) may identify patients with certain types of cancers who are more likely to respond to monoclonal antibodies that block the PD-1/PD-L1 pathway. There are two FDA-approved diagnostics for PD-L1 immunohistochemistry testing to guide therapeutic decision-making for patients with non-small cell lung cancer or melanoma who are candidates to receive anti-PD-1 therapies [13]. In addition, the FDA also very recently approved the use of the Ventana PD-L1 assay to the atezolizumab approved for use in advanced metastatic urothelial cancer (http://www.fda.gov/NewsEvents/Newsroom/PressAnnouncements/ucm501762.htm).

More progress with biomarkers of response are crucial, given the cost of immunotherapeutic agents and need to personalize and match likelihood of clinical benefit with the appropriate treatment. For example, the demonstration that PD-L1 expression may improve the likelihood of successful immunotherapy has provided insight into using other agents that may increase PD-L1 expression as a method to improve PD-1 blockade and is an active area of current investigation. Limited access to biospecimens that may otherwise be discarded or placed in storage seems counterproductive to the research mission. Most importantly, the mission of personalized medicine, the aim of which is to treat each patient with the drugs he or she is most likely to respond to, sparing unnecessary side effects and reducing cost, requires by definition access to biospecimens. At a time when technologies are rapidly evolving and considerable information can be learned from even small samples, it is unadvisable to perform a clinical trial without collecting the samples that will allow the investigators to understand why patients respond or do not respond, a necessary step for making progress in improving treatments.

SITC values its responsibility to patients, and society at large, to help foster the ethical conduct of clinical research in order to improve the lives of patients with cancer. We believe that continued research is essential to this process and must be conducted with appropriate regulatory oversight, institutional compliance and with respect for individual patients. While we welcome the timely review of the Common Rule and welcome a more comprehensive discussion with all stakeholders, we caution the sponsors of the proposed changes to carefully consider those measures that may inhibit investigation of correlative aspects of contemporary clinical trials through increased restrictions on access to biospecimens. The decision to donate such materials should remain with a well-informed, individual patient while recognizing that there may be real benefits to both patient and society. The ability to decline such participation should also remain with the donor. The progress of the past decade has been enormous and has been well served by informed interactions between medical experts, regulatory agencies and individual cancer patients. Continued dialogue should enhance clinical investigation allowing even further progress in the common goal of elimination the burden of cancer for patients and humankind.
